# Antenatal and histological diagnostics of cystic sacrococcygeal teratoma. Clinical case and literature review

**DOI:** 10.1515/crpm-2022-0025

**Published:** 2023-01-11

**Authors:** Egle Savukyne, Saule Krzconaviciute, Marija Vaitkeviciute, Egle Machtejeviene, Ieva Rubaviciute

**Affiliations:** Medical Academy, Department of Obstetrics and Gynaecology, Lithuanian University of Health Sciences, Kaunas, Lithuania; Medical Academy, Lithuanian University of Health Sciences, Kaunas, Lithuania; Medical Academy, Department of Pathological Anatomy, Lithuanian University of Health Sciences, Kaunas, Lithuania

**Keywords:** antenatal diagnostics, case report, cystic sacrococcygeal teratoma, fetal dysplasia, myelocystocele

## Abstract

**Objectives:**

The congenital embryonic tumor known as sacrococcygeal teratoma (SCT) affects 1 in 35.000–40.000 newborns and is more prevalent in female fetuses and neonates. A total of 25–50% of SCTs are diagnosed by an ultrasound (US) examination during the second trimester of pregnancy. Planning the manner of delivery, determining the risk of negative outcomes, and choosing treatment options depend on the results of antenatal differential diagnosis.

**Case presentation:**

This is a unique case of a 29-year-old second gravida, suspected of having a fetal sacrococcygeal dysplasia differentiable between Type 2 SCT and terminal myelocystocele. An MRI revealed no typical SCT changes, as a matter of course, the diagnosis of myelocystocele could not have been excluded. The results of the genetic examination allowed to exclude the chromosomal pathology. Punctuation of the external component of the formation and a cytological examination were suggested. Nevertheless, the patient and her partner refused further studies and insisted on the termination of pregnancy. Medical abortion was induced and histological findings confirmed fetal morphology to be mature SCT.

**Conclusions:**

Cystic sacrococcygeal teratoma is an unusual malformation of fetal development. In the antenatal period SCT is diagnosed based upon an ultrasound evaluation, an MRI, and a multidisciplinary assessment of clinical experts. Differential diagnosis based upon clinical imaging during the gestational period is elaborate. The final medical diagnosis needs to be verified by a histological evaluation of pathological tissue. An antenatal medical diagnosis of fetal dysplasia is considerable for the further prognosis of fetal and newborn development.

## Introduction

The Sacrococcygeal Teratoma (SCT) is an uncommon congenital germ cell tumor that affects 1 in 35.000–40.000 newborns and is more prevalent in female fetuses and neonates [1, 2]. The SCT consists of all three germ layers, hence, the structure of the tumor is polymorphic and includes tissue of various differentiation. Ordinarily the tumor is benign and is removed surgically, however, a recurrence of the tumor and its transformation into teratocarcinoma or yolk sac tumor is possible [3]. Altman et al. published the SCT classification that is currently applied. This classification describes teratomas according to their location (Table 1) [3].

**Table 1:** The classification of sacrococcygeal teratomas.

Types	Description
Type 1	– The majority of the formation is on the fetus’s outside (external component), while there may also be a tiny inside component. – The lowest risk of a recurrence of the cancer.
Type 2	– The formation consists of an external and an internal presacral component.
Type 3	– The formation is mostly on the fetus’s inside, in the fetal abdominal cavity, while there may also be a tiny outside component.
Type 4	– The formation is only on the fetus’s inside, in the presacral region. There is no external component. – The highest risk of a recurrence of the cancer.

A total of 25–50% of SCTs are diagnosed by an ultrasound (US) examination during the second trimester of pregnancy [1, 4, 5]. Mortality caused by SCT complications is three times higher if the diagnosis is made antenatally [6]. The scientific findings indicate that SCT in most cases is not associated with chromosomal abnormalities [6, 7]. However, there are few cases of SCT chromosomal pathologies in patients diagnosed antenatally, encasing partial chromosome 10q and partial chromosome 17p, partial chromosome 7q/trisomy 2p, and trisomy 1q [8], [9], [10].

A myelocystocele is a closed (skin-covered) spine fissure caused by central spinal canal cysts and herniated spinal cord sheaths [11]. Similarly to SCT, female fetuses are more frequently found to have this abnormality [12]. Some teratogens, including hydantoin, loperamide hydrochloride, and retinoic acid, have been identified as potential risk factors for myelocystocele, while the exact cause remains unknown [7]. Both fetal abnormalities are typically visible in an US screening. Fetal magnetic resonance imaging (MRI) is conducted for the purpose of differential diagnosis. In the case of myelocystocele, spinal dysraphia is noticeable, which is one of the primary criteria of visual differential diagnosis [13].

Planning the manner of delivery, determining the risk of negative outcomes, and choosing treatment options all depend on the results of antenatal differential diagnosis. If a teratoma is suspected, a multidisciplinary team of professionals, including obstetricians-gynecologists, neonatologists, pediatric neurosurgeons, pediatric surgeons, radiologists, and oncologists, have to determine the diagnosis and further treatment.

This article discusses a case of antenatal differential diagnosis between sacrococcygeal cystic teratoma and myelocystocele at 19 weeks of gestation, after an ultrasound evaluation revealed a thin-walled septated cystic formation consisting of external and internal components in the fetal sacrum region.

## Case presentation

**Figure 1:**
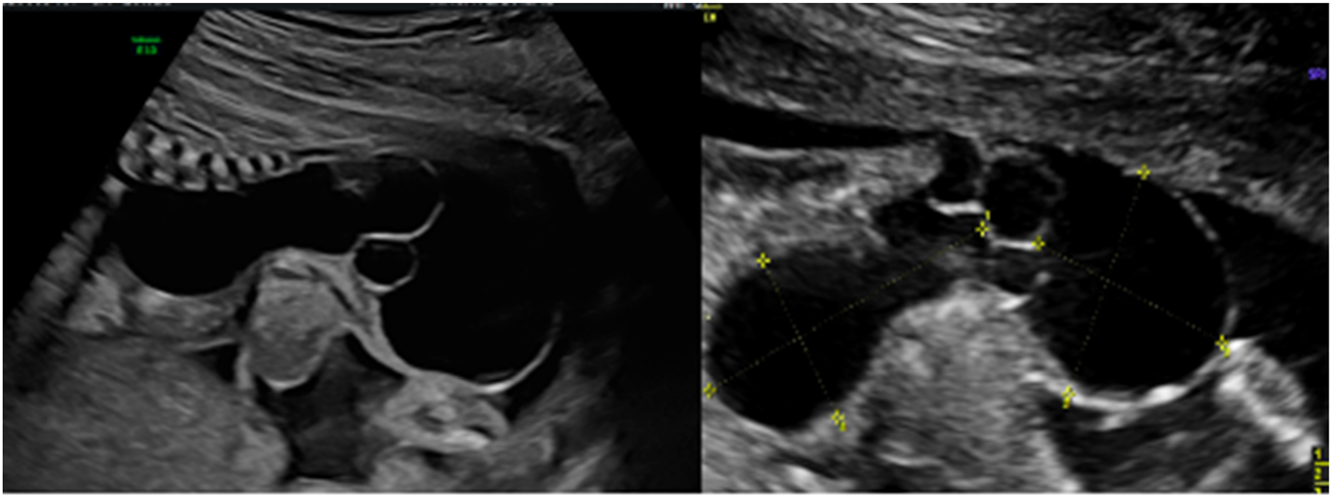
Fetal sacrococcygeal cystic formation at 19 weeks of gestation.

**Figure 2:**
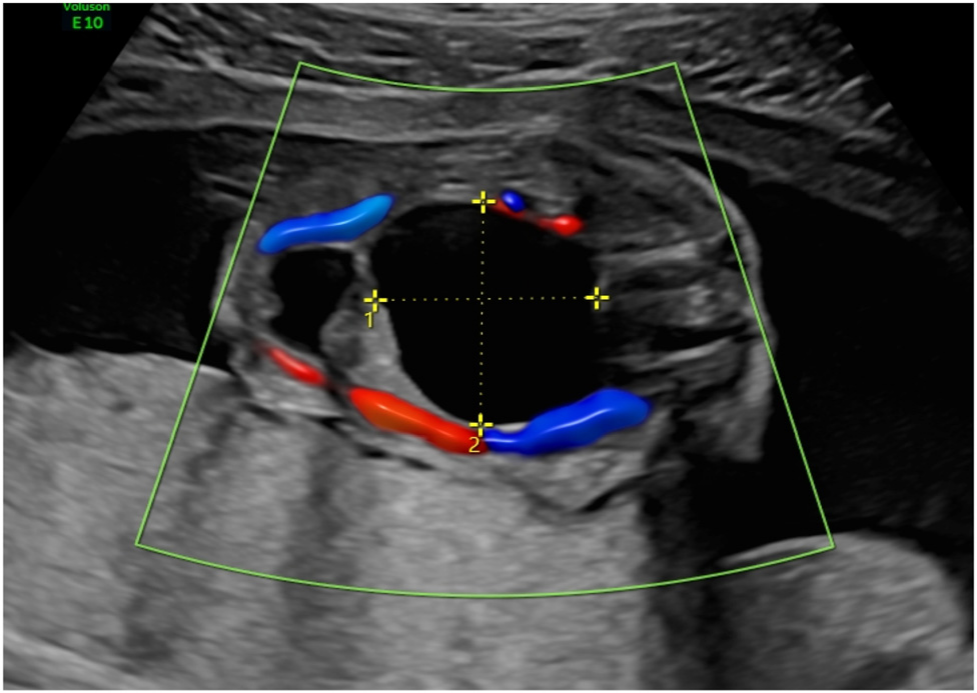
The internal component of the cystic formation and the urinary bladder.

A 29-year-old gravida 2, para 2 patient at 19 weeks of gestation was administered to the Hospital of the Lithuanian University of Health Sciences (LUHS) Kaunas Clinics, Outpatient department of Obstetrics and Gynaecology as a matter of urgency for prenatal ultrasound examination under a suspicion of fetal dysplasia. The patient and her partner had no comorbidities and no family history of hereditary diseases. The partner of the patient was a 33-year-old smoker male. Their first newborn was diagnosed with hypospadias, and the operation was performed at the age of 18 months. The patient was taking folic acid throughout the first trimester of this pregnancy. During the ultrasound screening biometric measurements of the fetus corresponded to the duration of gestation measured by the date of the last menstrual cycle (the menstrual cycle of a patient was regular). The fetal sacrum pathology was diagnosed – a thin-walled cystic formation with partitions in the sacrococcygeal region was visible during the examination. The external formation had a connection to the cystic structure in the fetal abdominal cavity. A clear solid component of the cyst and blood flow inside the cystic formation were not detected (Figures 1 and 2). The structures of the fetal brain were observed to be with no deviation.

The patient’s condition was discussed in a meeting of multidisciplinary medical specialists regarding congenital fetal pathology. Type 2 cystic SCT was identified as the most probable diagnosis. However, it had to be differentiated with terminal myelocystocele since the connection of the formation to the spinal cord remained unclear. A decision was made to perform a fetal MRI, diagnostic amniocentesis, and genetic examination. At the Hospital of the LUHS Kaunas Clinics, Genetics and Molecular Medicine Clinic, specific chromosomal markers located on the 13th, 18th, 21st, X, and Y chromosomes were examined by quantitative fluorescence PCR using the Devyser Complete v2 kit. The results of the study made it possible to exclude fetal pathology with regards to the irregular number of chromosomes.

**Figure 3:**
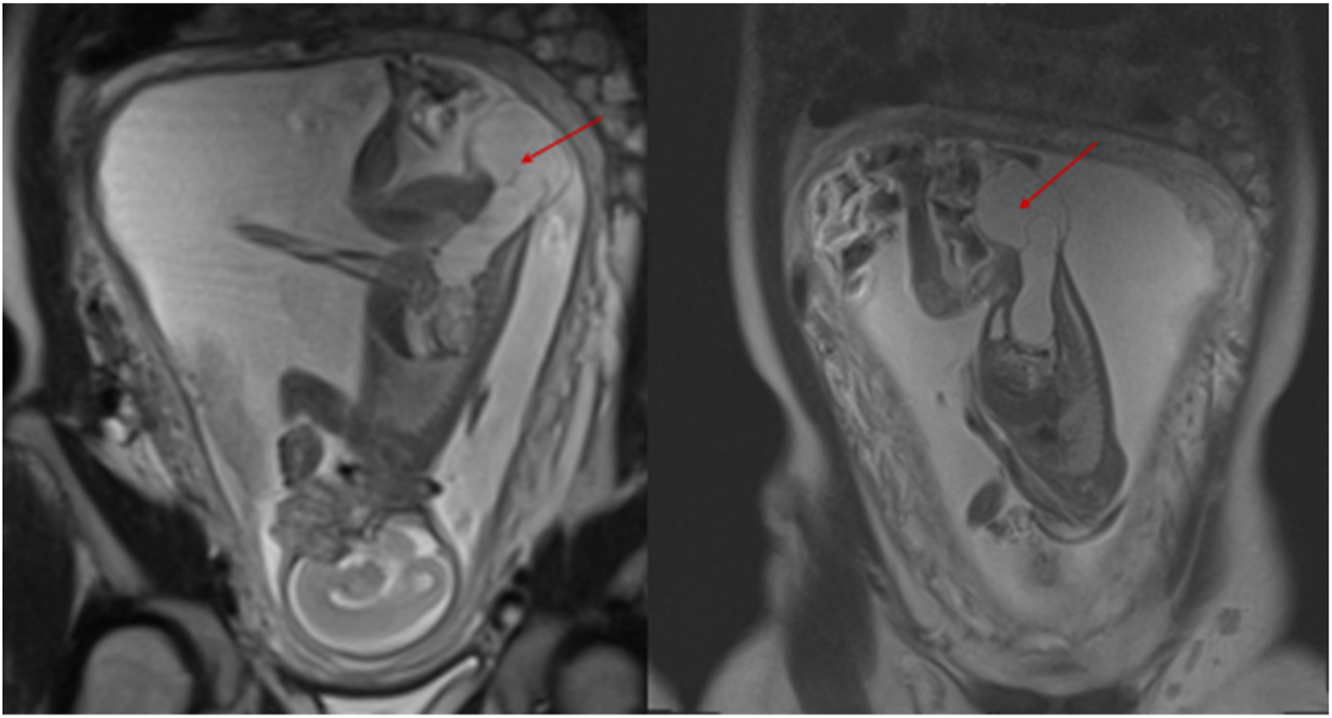
Fetal MRI of a cystic formation in the sacrococcygeal region.

During the fetal MRI examination, a cystic mass of large measurements (11.6 × 32.7 mm in the abdominal cavity, 40.7 × 34.7 mm in the sacrococcygeal region) was detected (Figure 3). The partitions were visible on the inside of the formation. On the fetal MRI, a narrow CSF band with the connection to the cystic mass and dura mater sac was suspected in the sacral region of the fetus. After a careful analysis of the scientific literature and the MRI results, it was decided that the mentioned changes are not typical for SCT and the diagnosis of myelocystocele cannot be excluded.

After the fetal MRI, the diagnosis remained differentiable between Type 2 SCT and terminal myelocystocele, thus, a second multidisciplinary medical consultation was organized at 21 weeks of gestation. Ultrasound and MRI images were analyzed and it was concluded that the antenatal diagnosis of fetal dysplasia was crucial for the future prognosis of the fetus, however, it remained ambigous. It was also unknown how much the cystic formation would grow in size during the remaining gestational period and what would be the size of the damage to the fetus. The patient and her partner were informed that the newborn would need at least two large-scale surgeries (removal of the external cystic formation and laparotomy for the removal of the internal cystic formation). During the consultation, it was concluded to offer the parents to punctuate the external component of the formation and perform a cytological examination of the contents of the cyst. Nevertheless, the patient and her partner refused further studies and insisted on the termination of pregnancy as soon as possible in consequence of the identified fetal dysplasia and possible negative outcomes.

The patient was consulted by a psychologist and a social worker. After the consultation she was hospitalized at the Hospital of the LUHS Kaunas Clinics, Obstetrics and Gynecology Clinic at 21 weeks of gestation. Stillbirth was induced with prostaglandins. The patient misscarried a 595 g male fetus with a large cystic mass in the sacroccocygeal region. The fetus, placenta, and amniotic sac were sent for histological examination.

**Figure 4:**
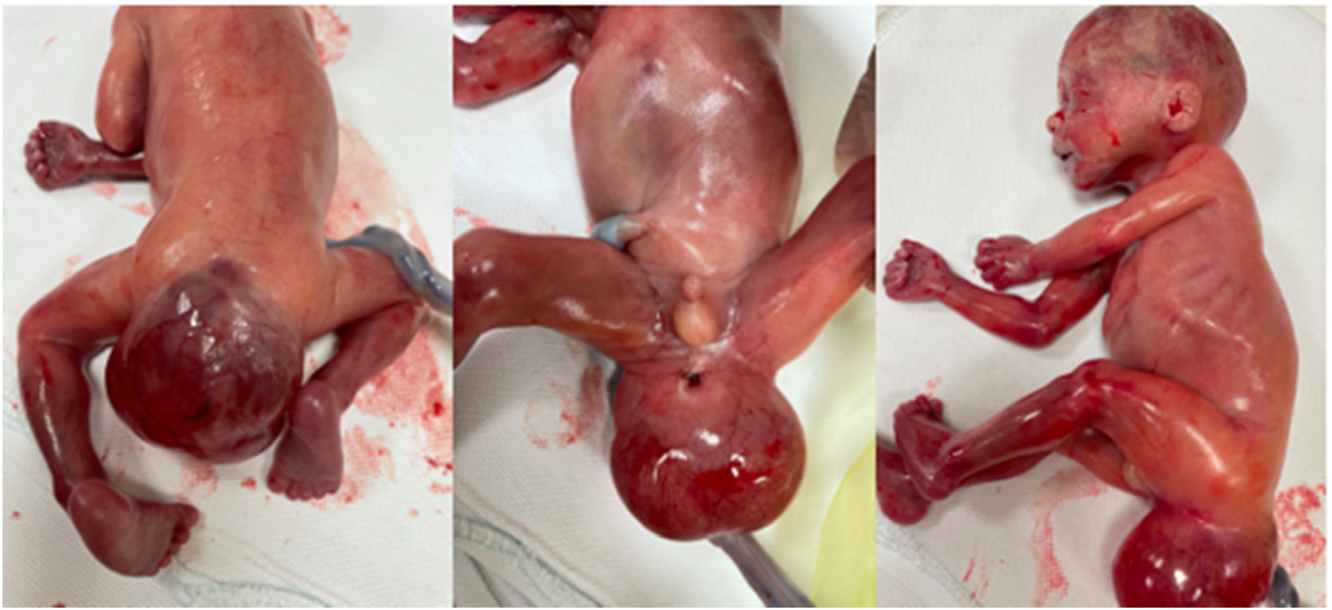
Macroscopic view of the formation located in the sacroccocygeal region.

**Figure 5:**
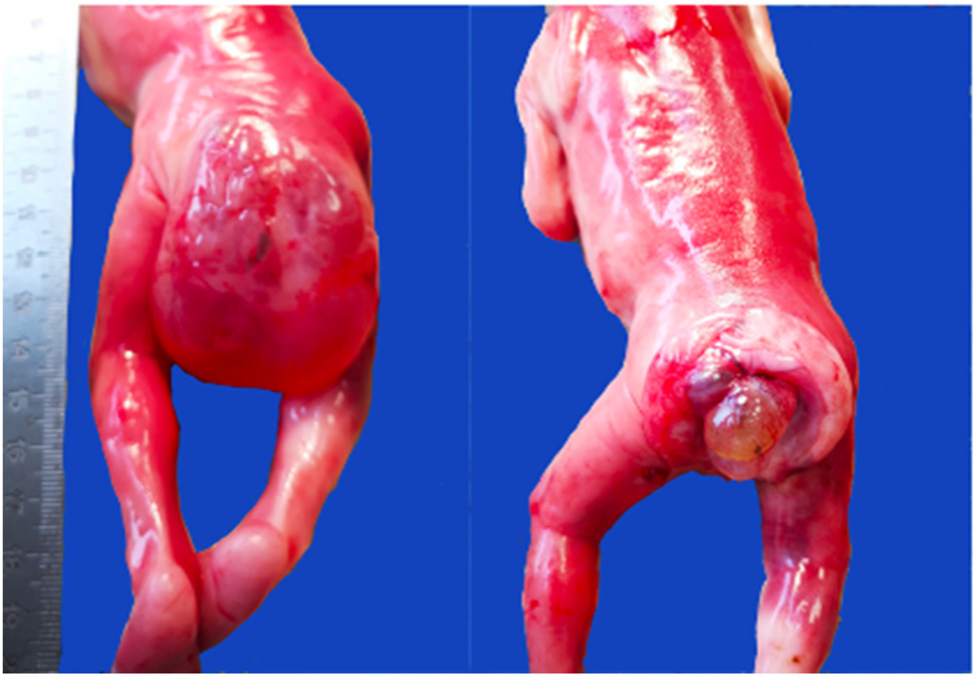
Multichambered cystic formation in the sacrococcygeal area.

The macroscopic examination revealed a cystic formation (50 × 45 × 50 mm) in the sacrococcygeal area (Figures 4 and 5). After opening the cystic formation, its wall was found to be up to 3 mm in thickness. A second multi-chambered cystic formation was of 15 mm diameter and was visible in the sacral region. The inner surface of the formation was smooth, the cysts were filled with clear serous fluid, and the cyst cavity had a connection to the pelvis. There was no connection to the spinal canal.

**Figure 6:**
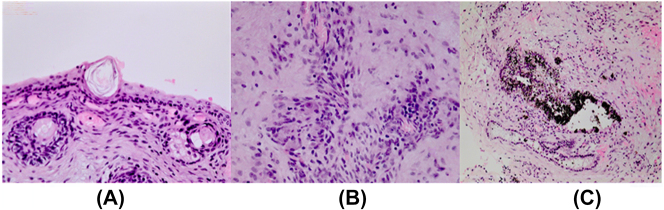
Histological examination of cyst tissue: A – Stratified squamous keratinized epithelium with dermal appendages. B – Structures of the vascular plexus. C – Retinal structures with black-brown pigment.

Microscopically, it was confirmed that the wall of the pathological structure was formed by the connective tissue. The cavity was lined from the inside by stratified squamous epithelium (Figure 6A) and foci of vascular plexus (Figure 6B). Skin appendages (Figure 6A), hair roots, and sebaceous glands were found in the wall of the formation, along with muscle, nerve, and retina-like tissue with black-brown pigment (Figure 6C). The placenta was found to be immature, corresponding to gestational age and with small ischemic foci. Fetal morphology was concluded to be mature sacrococcygeal cystic teratoma.

## Discussion

The antenatal differential diagnosis of sacrococcygeal teratoma is broad and includes terminal myelocystocele, meningocele, myelomeningocele, lipoma, hamartoma, lymphangioma, hemangioma, chordoma, and ependymoma [13]. The described clinical case emphasizes the difficulty of differentiating between SCT and terminal myelocystocele and establishing an accurate diagnosis antenatally. Other authors describe different SCT cases of delayed diagnosis which correlated to a complicated course of the disease [14], therefore, an early and accurate diagnosis is of crucial importance in SCT cases.

Studies conducted by different authors reveal that the primary medical diagnostic measures for differentiating the fetus’s pathological formation of the sacrococcygeal region and predicting the course of the condition are fetal and newborn ultrasound (US) and magnetic resonance imaging (MRI) [1, 7, 14, 15]. An MRI is particularly useful in the process of confirming the diagnosis and enables not only to differentiate but also to assess the size of the tumor and the prognosis of the disease. After birth, the localization of the tumor and the relationship with the surrounding structures is determined via an US and an MRI. A computer tomography (CT) may be additionally performed [13]. On US examination SCT is visible as an irregular thick-walled mass with cystic and solid tissue components [1, 6] while terminal myelocystocele reminds of a “cyst within a cyst” filled with cerebrospinal fluid [16] and an ependymal sac covering the lumbar vertebrae and sacrum is observed. In case of terminal myelocystocele on an US or an MRI, the posterior elements of the vertebral bodies are frequently wide, and a dilated central canal is recognized in the erupted sac [17]. To assess any abnormalities in the brain, spinal cord, spine, or lateral ventricles, it is crucial to perform a fetal US examination. Additionally, antenatal diagnosis is essential in selecting the best treatment strategies: in the case of cerebral cortex abnormalities or a high degree of kyphosis, it is recommended to perform fetal myelocystocele surgery before birth in order to improve the quality of life. In the presence of SCT, large cysts may be punctured and aspirated through the vagina or through the abdominal wall, in pursuance of reducing the size of the cyst before the delivery [18]. Maternal alpha-fetoprotein (AFP) concentration in serum or amniotic fluid is occasionally used for the screening of chromosomal abnormalities and assists in differentating SCT and terminal myelocystocele. In cases of a malignant SCT, AFP levels in amniotic fluid or maternal serum may be increased, however, terminal myelocystocele is not linked to the elevation in AFP [1, 13].

Yu et al. reported a case of suspected SCT at 30 weeks of gestation based on a presentation of polyhydramnios (240 mm) and a formation in the fetal sacrum region (100 × 100 × 60 mm). An MRI has not been performed due to the woman’s claustrophobia. After the delivery CT and MRI were performed on a newborn and the final diagnosis of terminal myelocystocele was established [13]. Clinical case of Takamiya et al. presents a challenging differential diagnosis of a tumor in the fetal sacrococcygeal region, which was detected during the US examination. An MRI has been performed and typical presentation of Type 2 SCT was revealed with a part of the tumor on the outside and another part in the pelvis and the presacral region of the fetus. These two cases with a very similar course of diagnosis confirm that in order to acquire an accurate diagnosis during the gestational period an MRI has to be performed [13] and the confirmed diagnosis can only be made after the delivery [19]. Although SCT and terminal myelocystocele may appear similar on the US examination (as well as in MRI in some cases) and the differential diagnostics are intricate, the outcomes and the prognosis of these two pathologies are dissimilar. The prognosis of a newborn diagnosed with SCT depends on the tumor growth, malignancy, and plausibility of radical surgical resection. Complications of SCT may include bleeding due to the rupture of a tumour and the risk of various pregnancy and delivery complications (polyhydramnios, premature birth, dystocia, etc.) increases [7]. In the case of SCT polyhydramnios develops due to arteriovenous effusions formation in the teratoma and can be visable during an US examination. As a result, it can cause fetal anemia and heart failure [13]. In 30% of SCT cases abnormalities in the development of various organ systems (neurological, digestive, urinary, skeleton, and cardiac) are present [17]. Rapid SCT growth may cause pressure on adjacent organs which results in malfunction of the fetal organism (e.g.: fecal and urine incontinence if pressure on the rectum or bladder is present) [13]. Patients diagnosed with terminal myelocystocele may be present with neurological, skeleton (lordosis, scoliosis, caudal dysgenesis or agenesis) or other (bladder exstrophy, omphalocele) disorders [17]. Accordingly, the impairment or loss of lower limb function, urination disorder or malabsorption may occur [14]. It has been established that in the presence of abdominal wall defects (e.g.: omphalocele), neurological defects are more frequently observed [14, 17].

A Caesarean section (CS) is recommended to reduce the risk of tumor rupture if a SCT of considerable measurment (>50 mm) is present. In the case of terminal myelocystocele a natural delivery is commonly possible, however a CS is occasionally recommended to avoid neurological tissue damage [13]. A surgical resection of teratoma within two months after the delivery is the main method of treatment [18, 19]. After a surgical operation is performed the concentration of AFP in the blood serum of the newborn can be monitored to assess the risk of maliganacy and recurrence of a teratoma [20]. In the case of terminal myelocystocele excision of the sac is highly recommended in order to prevent the occurance of neurological disorders and to improve life quality [7]. Early diagnosis of this condition along with an adequate treatment plan leads to a better prognosis and survival of the patients [21].

## Conclusions

Cystic sacrococcygeal teratoma is an unusual malformation of fetal development. In the antenatal period SCT is diagnosed based upon an ultrasound evaluation, an MRI, and a multidisciplinary assessment of clinical experts. Differential medical diagnosis based upon clinical imaging during the gestational period is elaborate. The final medical diagnosis needs to be verified by a histological evaluation of the pathological tissue. An antenatal medical diagnosis of fetal dysplasia is considerable for the further prognosis of fetal and newborn development.
